# P032: Clostridium difficile infection in Tenerife Canary Island, Spain

**DOI:** 10.1186/2047-2994-2-S1-P32

**Published:** 2013-06-20

**Authors:** M Hernández, M Ramos, M Lecuona

**Affiliations:** 1 Microbiología y Medicina Preventiva, Hospital Universitario de Canarias, La Laguna, Spain

## Introduction

*Clostridium difficile* infections(CDI) epidemiology has changed:elevation in rate and severity of infection and increase in disease among outpatients

## Objectives

Aim of study was to evaluate the epidemiology of CDI in North Tenerife Area.

## Methods

This is an epidemiological study performed in the Hospital Universitario de Canarias a tertiary care institution during 2011-2012.Studied population was outpatients or inpatients attended in this hospital with CDI suspected.Diagnostic procedures were based in GDH and later Toxin A/B detection as CDI confirmation in stool samples by EIA.Medical charts of patient were reviewed to collect: demographic variables, underlying diseases (diabetes(D), renal disease(RD),liver disease(LD),respiratory disease(ReD),cardiopathy(C),neoplasia(N)),>3 co-morbidities,inflammatory boweldisease(IBD),solidorgan transplant(OST), *immunocompromised states*(*IC*),*treatment previous*,*treatment of CDI*,*developed to Pseudo*-membranous *colitis*(*PMC*),*mortality due to CDI.The episodes were classificated as nosocomial*, *healthcare associated* (*HCA*),*community*, *indeterminate*, *and recurrence.*

## Results

In 2011/2012 a total 18/45 episodes (17/41 patients) were diagnosticated. 50/62% were man and 7/22 (39/49%),<65 years.HCA and nosocomial CDI incidence were:0,7/1,7 case/10^4^ patient–day.The services distribution was:Internal Medicine 6/12(33/27%), Nephrology 6/4(33/9%), Hematology 2/5(11/11%).Episodes:Community 4/7(22/15%), Nosocomial 14/27(78/60%), HCA 0/6(0/13%), Indeterminate 0/1(0/2%) and recurrences 0/4 (0/9%).In Nosocomial the time average between admission and CDI diagnostic was 10,6+9,1/24+29 d.Underlying diseases:C 10/9(55/20%),RD 5/10(27,7/22%),LD 2/4(11/9%), ReD 2/2(11/4%), N2/10(11/22%).>3 co-morbidities 3/3(17/7%). IBD 1/1(6/2%), OST 7/3(39/6,6%),IC 10/20(55/44%),previous treatment:Omeprazol 6/10(33/22%), Ranitidine 3/1(17/2%),Aciclovir 1/2(5,6/4,4%),Carbapenems 6/21(33/47%),Fluorquinolones 5/13(28/29%),Cephalosporins (3-4ª) 4/11 (22/24%),Vancomycin(VA) 3/6(17/13%), Amoxicillin-clavunate 2/7(11/16%).CDI Treatment:Metronidazole 18/39(100/87%), VA 3/9 (17/20%), developed PMC 2/5(11/11%), death for CDI 1/0.

## Conclusion

In our hospital there has been an increase in nosocomial CDI adquisition overtime and a high percentage in young patients.At 2012 OST patients declined and HCA episodes were increased thus we observed that CDI is not confined to hospitals.

## Disclosure of interest

None declared

